# Distinct responses of soil respiration to experimental litter manipulation in temperate woodland and tropical forest

**DOI:** 10.1002/ece3.3945

**Published:** 2018-03-13

**Authors:** Laëtitia M. Bréchet, Luis Lopez‐Sangil, Charles George, Ali J. Birkett, Catherine Baxendale, Biancolini Castro Trujillo, Emma J. Sayer

**Affiliations:** ^1^ Department of Biology Plants and Ecosystems (PLECO) research group in the research Centre of Excellence: “Global Change Ecology” University of Antwerp Wilrijk Belgium; ^2^ Lancaster Environment Centre Lancaster University Lancaster UK; ^3^ Centre for Ecology and Hydrology Wallingford UK; ^4^ Smithsonian Tropical Research Institute Panama City Panama; ^5^ School of Environment, Earth and Ecosystem Sciences The Open University Milton Keynes UK; ^6^ Teagasc Environmental Research Centre, Johnstown Castle Co. Wexford Ireland

**Keywords:** fine root biomass, forest ecosystems, litter manipulation, microbial biomass, priming effects, soil carbon dynamics

## Abstract

Global change is affecting primary productivity in forests worldwide, and this, in turn, will alter long‐term carbon (C) sequestration in wooded ecosystems. On one hand, increased primary productivity, for example, in response to elevated atmospheric carbon dioxide (CO_2_), can result in greater inputs of organic matter to the soil, which could increase C sequestration belowground. On other hand, many of the interactions between plants and microorganisms that determine soil C dynamics are poorly characterized, and additional inputs of plant material, such as leaf litter, can result in the mineralization of soil organic matter, and the release of soil C as CO_2_ during so‐called “priming effects”. Until now, very few studies made direct comparison of changes in soil C dynamics in response to altered plant inputs in different wooded ecosystems. We addressed this with a cross‐continental study with litter removal and addition treatments in a temperate woodland (Wytham Woods) and lowland tropical forest (Gigante forest) to compare the consequences of increased litterfall on soil respiration in two distinct wooded ecosystems. Mean soil respiration was almost twice as high at Gigante (5.0 μmol CO_2_ m^−2^ s^−1^) than at Wytham (2.7 μmol CO_2_ m^−2^ s^−1^) but surprisingly, litter manipulation treatments had a greater and more immediate effect on soil respiration at Wytham. We measured a 30% increase in soil respiration in response to litter addition treatments at Wytham, compared to a 10% increase at Gigante. Importantly, despite higher soil respiration rates at Gigante, priming effects were stronger and more consistent at Wytham. Our results suggest that in situ priming effects in wooded ecosystems track seasonality in litterfall and soil respiration but the amount of soil C released by priming is not proportional to rates of soil respiration. Instead, priming effects may be promoted by larger inputs of organic matter combined with slower turnover rates.

## 1 INTRODUCTION

Forest ecosystems play a crucial role in the global carbon (C) cycle: They represent the largest terrestrial C stock, because they cover 40% of the total land surface area (Jobbagy & Jackson, 2000), contain 82%–86% of the global aboveground biomass C (Dixon et al., 1994), and regulate a major exchange of C with the atmosphere through photosynthetic uptake and respiration (Malhi, Baldocchi, & Jarvis, 1999). Forest soils are particularly important in the global C balance, as much of the C stored in soils is thought to be relatively stable (Schlesinger, 1977). As much as 63% of the C stored in temperate forests is contained in soil organic matter, and even in the tropics, forest C storage is more or less equally partitioned between soil and aboveground biomass (Dixon et al., 1994). The quantity and quality of plant inputs to the soil (i.e., plant litter and root products) are the key drivers of organic matter turnover and residence times as they influence the amount and stability of soil organic C by regulating microbial decomposition processes (De Graaff, Classen, Castro, & Schadt, 2010). In turn, microbial mineralization of organic matter regulates the amounts of nutrients available for plant growth. Thus, interactions between plants and soil organisms influence a large number of ecosystem processes and play a key role in C cycling (Van der Heijden, Bardgett, & Van Straalen, 2008). Plant–soil interactions have gained considerable attention in the past few years because, they are likely to be influenced by climate changes such as rising temperature and altered precipitation patterns (De Vries et al., 2012; Van der Putten et al., 2013) but despite their importance in ecosystem C dynamics, we still lack a detailed understanding of many plant–soil interactions (Van der Putten et al., 2013). Importantly, a recent meta‐analysis revealed that the soil C turnover increases more rapidly in response to additional litter inputs than soil carbon (C) concentrations, which suggests that enhanced plant productivity under global change will not necessarily produce a corresponding increase in soil C storage (Xu, Liu, & Sayer, 2013). Given the importance of forests as major sinks or sources of atmospheric carbon dioxide (CO_2_), changes in plant‐soil interactions in forests could significantly affect the global C balance.

The “priming effect” is a particularly complex and poorly understood plant‐soil interaction, which could play an important role in soil C dynamics under climate change. Priming effects occur when a moderate increase in the input of fresh organic matter to the soil stimulates the microbial decomposition of older, stored soil C (Bingeman, Varner, & Martin, 1953; Kuzyakov, Friedel, & Stahr, 2000). As soil C is released as CO_2_ during the mineralization of soil organic matter, priming effects are often measured as a disproportionate increase in soil respiration. As our understanding of priming effects is mainly based on laboratory studies, in situ studies of this phenomenon are underrepresented, and the results are not always consistent across sites. Nonetheless, priming effects have been recognized as a key mechanism affecting soil C storage in long‐term free‐air CO_2_ enrichment (FACE) experiments in a range of forests, resulting in a smaller net gain in soil C storage or even a net loss, despite increased plant productivity and plant‐derived C inputs to the soil (Allen et al., 2000; Billings, Lichter, Ziegler, Hungate, & Richter, 2010; Hoosbeek & Scarascia‐Mugnozza, 2009; and Trueman & Gonzalez‐Meler, 2005). Litter manipulation experiments in both temperate (Crow et al., 2009; Sulzman, Brant, Bowden, & Lajtha, 2005) and tropical forests (Sayer, Heard, Grant, Marthews, & Tanner, 2011; Sayer, Powers, & Tanner, 2007) demonstrated that increased litter inputs released substantial amounts of C from the soil via priming effects and increased frequency of extreme weather events, such as storms and droughts, can cause large pulses of litter inputs within very short periods of time. However, although increased inputs of plant‐derived C could conceivably cause priming effects under a range of different environmental change scenarios, there is still great uncertainty about the relevance of priming effects in situ (Kuzyakov, 2010).

Comparative field experiments are key to identifying general principles and controls on soil C release by priming across different ecosystems. Although there are many individual studies of soil respiration at different sites worldwide, there are currently no large‐scale in situ studies of priming effects comparing different sites, and even in vitro experiments evaluating the influence of soil type are rare (but see Rasmussen, Southard, & Horwath, 2008; Nottingham, Turner, Chamberlain, Stott, & Tanner, 2012; Hamer & Marschner, 2005). Consequently, we have a severely limited understanding of the real‐world relevance of priming effects, and we know little about how their occurrence is influenced by, for example, seasonality of litterfall, temperature, and precipitation. We aimed to address this by performing a cross‐continental study to assess the response of soil C dynamics to experimental litter addition and litter removal in a temperate woodland and a tropical forest. We measured soil respiration (soil CO_2_ efflux) to identify general patterns across two distinct forest ecosystems and we compared priming effects in response to increased litter inputs between sites. Specifically, we tested the following hypotheses: (1) The response of soil respiration to litter manipulation treatments will be strongly influenced by the main constraint on decomposition at each site: soil temperature in the temperate woodland and soil water content at the tropical site. (2) As soil C turnover is faster in the tropics than in the temperate zone, priming effects will be greater in the tropical forest. (3) Given that litterfall is strongly seasonal, priming effects in response to increased litter inputs will track the seasonality of litterfall.

## 2 MATERIALS AND METHODS

### 2.1 Study sites

To compare the response of soil respiration to altered plant inputs in two distinct wooded ecosystems, we established parallel litter manipulation experiments in temperate woodland and tropical forest. The specific study sites were ideal for a direct comparison because, despite differences in climate, vegetation and soil type, the mineral soils at both sites had a total organic C content of c. 4.4%, total nitrogen (N) content of c. 0.5%, and a soil pH of c. 6.0 at 0–10 cm depth. Furthermore, there was an abrupt transition from organic surface horizons (L and F layers) to mineral soil, with no well‐developed humus (H) layer at either site.

The temperate site was located in old (c. 120 years) mixed deciduous temperate woodland in Wytham Woods, Oxfordshire, UK (51°46′42″N, 1°19′42″W; henceforth “Wytham”). The canopy layer is mainly dominated by a mixture of sycamore (*Acer pseudoplatanus* L.), ash (*Fraxinus excelsior* L.) and occasionally pedunculate oak (*Quercus robur* L.), and the sub‐canopy by hawthorn (*Crataegus monogyna* L.), and common hazel (*Corylus avellana* L.). The soil is a base‐rich clay loam classified as stagni‐vertic cambisol (FAO/WRB classification; Beard, 1993; IUSS Working Group 2006). The climate is temperate, with a mean annual air temperature of 10 ± 0.1°C and mean annual precipitation of 714 ± 29 mm (data from the Wytham weather station from 1993–2011; UK Environmental Change Network). The study site has had no silvicultural management for at least 40 years (Fenn, Malhi, Morecroft, Lloyd, & Thomas, 2015).

The tropical site was located on the Gigante Peninsula of the Barro Colorado Nature Monument in Panama, Central America (9°06′N, 79°54′W; henceforth “Gigante”). The vegetation is mature semi‐deciduous lowland tropical forest, which is at least 200 years old (Wright et al., 2011), and trees with trunk diameter at breast height (DBH) ≥10 cm are dominated by three families: Arecaceae (8%), Burseraceae (11%), and Olacaceae (12%; from study site inventory). The soil is a clay‐rich oxisol, with low extractable concentrations of phosphorus and potassium, but high base saturation and cation exchange capacity (Cavalier, 1992; Yavitt et al., 2009; ). The mean annual temperature on nearby Barro Colorado Island (c. 5 km from the study site) is 27°C and mean annual rainfall is 2,600 mm, with only c. 10% falling during the dry season from December to April (Windsor, 1990).

Our experimental design is based on an existing long‐term litter manipulation experiment at Gigante (Sayer, Tanner, & Cheesman, 2006; Sayer et al., 2007), but to enable a direct comparison between sites, we established 15 new experimental plots in five replicate blocks at each site in 2013. Each plot measured 25‐m × 25‐m and was trenched to c. 0.5‐m depth to minimize the transfer of nutrients and water via roots and hyphal networks; the trenches were lined with plastic and backfilled. To reduce trenching effects, a 5‐m buffer was left around the inside of the trenches, resulting in a measurement plot size of 15‐m × 15‐m. Starting in December 2013, all litter, including small branches (<1‐cm), was removed from five litter removal plots (L−) and immediately spread over five litter addition plots (L+), leaving five plots as undisturbed controls (CT). To account for differences in forest productivity and litterfall between study sites, the L− and L+ treatments were carried out monthly at Gigante and twice a year during the main period of litterfall at Wytham (October–January).

To estimate monthly litterfall, four litter traps were placed randomly in each plot; the frame of the traps measured 70.7‐cm × 70.7‐cm and was mounted c. 70‐cm above the soil surface. Litter samples, excluding woody litter with a diameter >2 cm, were collected on the last Thursday of every month, dried to constant weight at 60°C and weighed.

### 2.2 Soil respiration measurements

To measure soil respiration, we installed four permanent soil collars in each plot. The collars were made of PVC tubes (20‐cm inner diameter and 12‐cm height), which were sunk into the soil to 2–3 cm depth. The collars were installed c. 7.5 m from the center of each side of the plots at least 4 weeks before measurements began.

We measured soil respiration monthly at each site from December 2013 to December 2015, then every one to two months until October 2016 (Wytham) or November 2016 (Gigante). Before each measurement, we carefully removed as much litter and organic material as possible from the inside of the collars without disturbing the underlying mineral soil and replaced it once the measurement was completed. Soil respiration was measured using an automated soil CO_2_ flux system (Li‐8100; LiCor Biosciences, Lincoln, USA) consisting of an infrared gas analyzer connected to a 20‐cm survey chamber. The CO_2_ concentration in the chamber was measured and logged every second for 2 min and CO_2_ efflux was calculated by exponential or linear regression of the CO_2_ concentration over time. During each respiration measurement, we took three measurements of soil water content using a ThetaProbe (0–6 cm depth; Delta‐T Device, Cambridge, UK), and measured soil temperature at 0–10 cm depth with a temperature probe; all measurements were taken within c. 0.5‐m of the soil collar.

### 2.3 Data analysis

All statistical analyzes were conducted using SAS version 9.3 (SAS Institute, USA). Values of soil respiration that were exceptionally high (>10 μmol CO_2_ m^−2^ s^−1^) or low (<1 μmol CO_2_ m^−2^ s^−1^) were considered outliers (c. 6% and 0.4% of all measurements from Gigante and Wytham, respectively) and were removed prior to statistical analysis. We used mean values per plot for all variables and data were log‐transformed where necessary to meet the assumptions of linear models. To directly compare the effect of litter manipulation treatments between sites, we calculated the log response ratios (RR) for soil respiration as RRx = ln(Rx/Rc), where Rx is the measured value of the response variable in a given experimental treatment and Rc is the control value (Hedges, Gurevitch, & Curtis, 1999). Response ratios greater than zero indicate positive effects of litter treatment on soil respiration whereas values lower than zero represent negative responses.

Linear and nonlinear regression models were used to investigate the relationships between soil respiration and soil water content or soil temperature. First, we determined the functions to describe the relationships between soil respiration and soil temperature or soil water content for each site. For Wytham, the relationship between soil respiration and temperature was best described by:

(1) $$\text{SR}=ae^{\left(bT\right)}$$

where SR is soil respiration (μmol CO_2_ m^−2^ s^−1^), *T* is soil temperature (°C), and *a* and *b* are constants. The *Q*_10_ value for the response of soil respiration to a 10°C change in temperature was then calculated as:

(2) $$Q_{10}=e^{10b}$$

The relationship between SR and soil water content differed between the two sites; for Gigante, the relationship was best described by:

(3) $$\text{SR}=aHv^{2}+bHv+c$$

whereas for Wytham, the relationship was described by:

(4) SR=aHv+b

where *Hv* is the soil water content (%) and *a*,* b,* and *c* the constants fitted to each regression model.

We asked whether the response of soil respiration to litter manipulation treatments varied among sites. First, we assessed the relationship between litterfall and soil respiration, taking seasonality and decomposition rates into account using a stepwise approach to identify the time‐lag between monthly litterfall rates and corresponding changes in soil respiration for each site. We then used nested linear mixed effect models to assess the influence of treatment, site, and monthly litterfall on soil respiration. Litter treatment, site, litterfall (with a time‐lag) and their interaction were included as fixed effects, block and time were random effects.

Although differences in fine root or microbial biomass could explain changes in soil respiration, we observed no changes in soil microbial biomass or fine root biomass among treatments at either site (Table S2). We, therefore, estimated respiration from decomposition of recently‐incorporated organic matter (litter‐derived soil respiration; SR_LITTER_) from the difference in soil respiration between the CT and L− plots. We then calculated priming effects (PE) for each block and month in which the increase in soil respiration with litter addition exceeded SR_LITTER_ as:

(5) $$\text{PE}=\left({\text{SR}}_{L+}-{\text{SR}}_{\mathrm{CT}}\right)-\left({\text{SR}}_{\mathrm{CT}}-{\text{SR}}_{L-}\right)$$

where SR_L+_, SR_CT,_ and SR_L−_ are monthly means of soil respiration in μmol CO_2_ m^−2^ s^−1^, in the litter addition, control, and litter removal plots, respectively. Finally, we examined the influence of site, soil temperature, and soil water content on priming effects using nested linear mixed‐effects models in which site, soil temperature, soil water content, and their interaction were included as fixed effects, and block and time were included as random effects.

## 3 RESULTS

### 3.1 Site and litter manipulation effects on soil respiration

**Figure 1:**
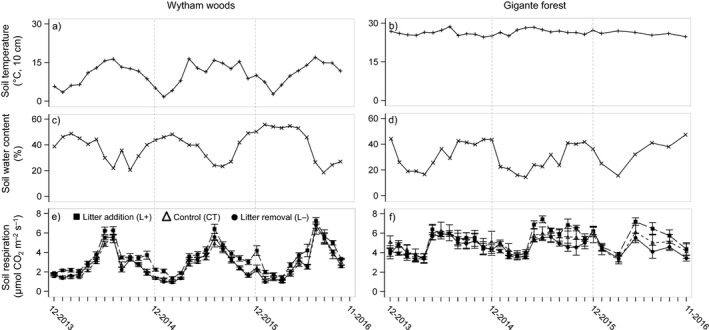
Dynamics of (a and b) soil temperature, (c and d) soil water content, and (e and f) soil respiration in plots exposed to different litter manipulation treatments in temperate woodland (Wytham Woods) in the UK (left‐hand panels), and lowland tropical forest (Gigante Peninsula), in Panama (right‐hand panels) from December 2013 to November 2016; means across all treatments are shown except for soil respiration, where means ± standard errors are shown for *n *=* *5 per treatment and site

Although mean soil respiration over the study period was almost twice as high at Gigante (5.0 ± 0.87 μmol CO_2_ m^−2^ s^−1^) as at Wytham (2.7 ± 1.39 μmol CO_2_ m^−2^ s^−1^; Figure 1e,f), the litter manipulation treatments had a greater effect on soil respiration at Wytham (Figure 3c,d). At Wytham, soil respiration increased significantly in the L+ plots (*t *=* *5.37; *p *<* *.001; Figure 1e) almost immediately after the treatments were applied, and remained c. 30% higher than the controls during the 3 years of the study (Figure 1e). By contrast, at Gigante, there was no effect of either litter manipulation treatment for the first year but from 2015, mean soil respiration was c. 10% higher in the L+ plots compared to the controls (*t *=* *3.36; *p *<* *.05; Figure 1f). The best model for soil respiration included site, litter treatment, and their interaction (χ^2^ = 372.62, *p *<* *.001 and *p *<* *.001 for litter treatment and site, respectively; Table 1). From 2015 onwards, litter addition had a greater overall effect on soil respiration than litter removal, and the mean relative increase in soil respiration at Wytham (30%) was greater than at Gigante (10%). By contrast, soil respiration in the L− plots did not differ significantly from the controls at either site.

**Table 1:** Results of linear mixed‐effects model with soil respiration (log‐transformed) as the response variable, litter treatment, site, litterfall, and their interaction as fixed effects, block and month as random effects (Model 1: AIC = 802.9; χ^2^ = 372.62; *n* = 927; Model 2: AIC = 536.4; χ^2^ = 187.51; *n* = 926 for the all study years)

Source of variation	Soil respiration	
	*F*	*p*
Model 1		
Site	786.40	<.001
Treatment	42.77	<.001
Site × treatment	10.74	<.001
Model 2		
Site	277.35	<.001
Treatment	44.62	<.001
Litterfall	34.15	<.001
Site × litterfall	38.57	<.001

### 3.2 Seasonality and links between soil respiration and litterfall

**Figure 2:**
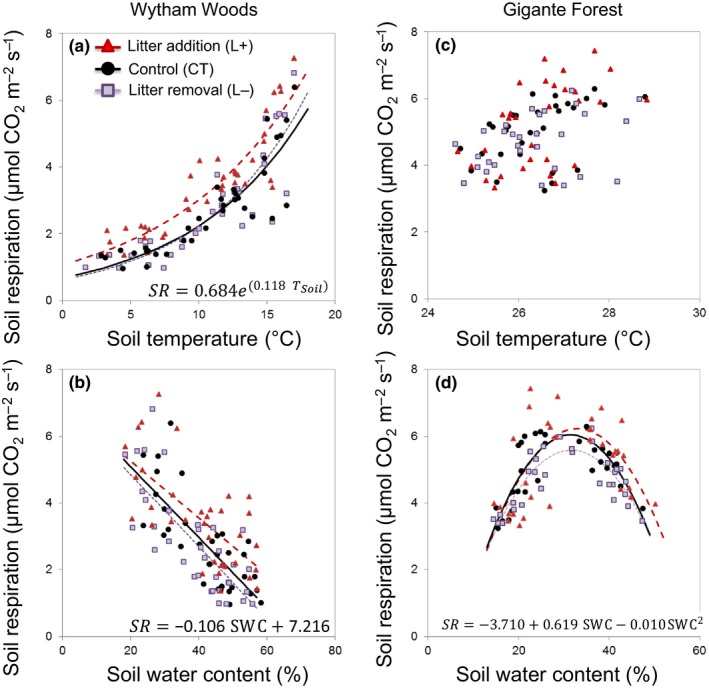
Relationship between soil respiration and soil temperature or soil water content in litter manipulation plots in (a and b) a temperate deciduous woodland in the UK (Wytham Woods) and (c and d) lowland tropical forest, in Panama (Gigante) from December 2013 to November 2016; each point represents the mean of four measurements per plot for *n *=* *5 plots per treatment. Equations and their parameters are shown for the CT plots

Soil respiration had a similar seasonal pattern at both sites, with low rates in winter (1.6 ± 0.5 μmol CO_2_ m^−2^ s^−1^) and in the dry season (4.5 ± 1.0 μmol CO_2_ m^−2^ s^−1^) and higher rates in summer (3.6 ± 1.3 μmol CO_2_ m^−2^ s^−1^) and in the wet season (5.4 ± 0.6 μmol CO_2_ m^−2^ s^−1^) at Wytham and Gigante, respectively (Figure 1e,f). However, the seasonality of soil respiration was underpinned by distinct relationships with soil temperature and water content. At Wytham, soil temperature and water content varied strongly throughout the year; the relationship between soil respiration and soil temperature was best described by a positive exponential function (Figure 2a; Table S1), whereas the relationship between soil respiration and soil water content was described by a negative linear function (Figure 2b; Table S1). At Gigante, soil temperature was constant throughout the study period, with <0.5°C difference between the wet and dry seasons, whereas soil water content was >44% higher in the wet season. Accordingly, there was no significant relationship between soil respiration and soil temperature (Figure 2c; Table S1) but the effect of soil water content on soil respiration was significant (*F *=* *7.68; *p *<* *.01) and best predicted by a quadratic function (Figure 2d; Table S1).

The litter treatments had only a minor influence on soil water content and soil temperature, and the effect of treatment varied by site. At Wytham, mean soil water content was 5% lower in the L+ and 8% lower in the L− treatments compared to the CT plots, but the difference was not significant. Although soil temperature did not differ among litter treatments, the higher *Q*_10_ value in the L− treatment at Wytham indicated that soil respiration was more sensitive to changes in soil temperature when the litter layer was removed (Table S1). At Gigante, although differences were not significant, soil water content was 4% and 7% higher in the L+ and L− treatments compared to the CT plots, and soil water content explained more of the variation in soil respiration in the L− plots (*R* ^2^ = .74; Table S1).

**Figure 3:**
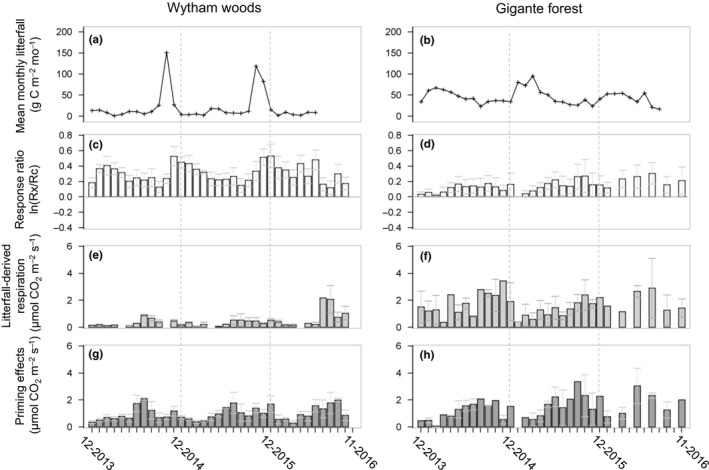
Dynamics of changes in (a and b) litterfall biomass carbon in control plots, (c and d) soil respiration in response to litter addition, (e and f) litter‐derived soil respiration and, (g and h) priming effect due to litterfall from December 2013 and November 2016 for the Wytham Woods temperate forest, in England (on the left), and from January 2015 and November 2016 for the Gigante tropical forests, in Panama (on the right); soil respiration at Gigante was only measured every 2 months in the final year of the study

Litterfall was also highly seasonal at both sites (Figure 3a,b). Peak litterfall at Wytham occurred at the end of the growing season in October and November, whereas at Gigante, the highest rates of litterfall occurred at the start of the dry season in January. Over the study period, mean annual litterfall at Wytham (2.43 ± 0.76 Mg C ha^−1^ year^−1^) was almost 50% lower than mean annual litterfall at Gigante (5.47 ± 0.38 Mg C ha^−1^ year^−1^), litter‐derived respiration in the mineral soil was highly variable over the time at both sites (Figure 3a,b,e,f) and the mean contribution of litter to annual soil respiration was much lower in Wytham compared to Gigante (19% and 29%, respectively). Despite the minor overall contribution of litter to annual total soil respiration, the models describing seasonal variation in soil respiration were improved when monthly litterfall was included with a time lag of 8 months for Wytham and 4 months for Gigante (χ^2^ = 187.51, *p *<* *.001 for litter treatment, site, and time‐lagged litterfall, respectively; *n *=* *926; Table 1), demonstrating the importance of litterfall and decomposition in seasonal patterns of soil respiration.

### 3.3 Soil C release by priming effects in response to litter addition

As there was no significant response of soil respiration to litter addition at Gigante during 2014, we calculated priming effects for 2015–2016 at both sites to ensure a direct comparison. The best model for soil C priming included site, soil temperature, and their interaction (χ^2^ = 75.13; *p *<* *.001; *n *=* *200). At Wytham, 85% of all measurements showed a greater increase in soil respiration in the L+ plots than would be expected from the added litter, indicating additional release of soil C by priming effects. By contrast, at Gigante, we only observed priming effects during 58% of all measurements. Hence, although soil respiration rates were higher at Gigante, the mean soil C release attributed to priming was greater at Wytham (0.88 μmol CO_2_ m^−2^ s^−1^ at Wytham and 0.73 μmol CO_2_ m^−2^ s^−1^ at Gigante), indicating that the stronger response of soil respiration to litter addition at Wytham was largely due to priming effects.

Soil C release by priming showed seasonal patterns at both sites, which largely tracked changes in soil respiration. The largest priming effects at Wytham occurred c. 8 months after the application of litter treatments each year, whereas at Gigante, soil C release by priming was generally low during the dry season but there was no clear peak during the rainy season (Figure 3g,h).

## 4 DISCUSSION

We compared the response of soil respiration to 3 years of litter manipulation treatments in two distinct forest ecosystems: temperate woodland in the UK and tropical forest in Panama. Increased litter inputs to soils enhanced soil respiration at both sites, and for the majority of measurements, the increase in soil respiration in the L+ plots was greater than expected. The extra release of CO_2_ could not be attributed to changes in microbial biomass C or increased root biomass (Table S2), which suggests soil C release by priming effects (Sayer et al., 2007, 2011) as a result of altered microbial activity or community composition (Kuzyakov, 2010).

As hypothesized, we found consistent patterns in soil respiration in response to the experimental treatments across continents, with links between priming effects and litterfall seasonality. However, we also observed important differences in the magnitude and occurrence of priming effects between temperate and tropical ecosystems. We expected greater soil C release by priming effects with high C turnover in the tropical forest at Gigante, but instead, we observed a greater effect of litter addition and magnitude of priming effects in the temperate woodland at Wytham.

### 4.1 Distinct dominant abiotic controls result in similar seasonal patterns of soil respiration

Both forest sites showed a similar seasonal pattern of soil respiration, with highest rates during the growing seasons from April–September at Wytham and May–October (rainy season) at Gigante, and the lowest rates during winter (October–March) at Wytham and the dry season (November–April) at Gigante (Figure 1). Despite these similarities, the underlying controls of the seasonal pattern differed between sites. Soil temperature and soil water content are the main drivers of soil respiration on a global scale (Raich & Schlesinger, 1992) and the patterns we observed across all treatments reflect temperature constraints on soil CO_2_ efflux during winter at Wytham (Figure 2a), but constraints at both low and high soil water content at Gigante (Figure 2d). These constraints were also apparent in seasonal changes in the magnitude of the soil respiration response to litter manipulation treatments (Figures 1e,f and 3c,d).

As expected, soil respiration at Wytham increased with temperature in all treatments. However, although the sensitivity of soil respiration to changes in temperature (*Q*_10_) in the CT plots was similar to values reported for other temperate forests (Kicklighter et al., 1994), it is noteworthy that the *Q*_10_ was higher in the L− and lower in the L+ treatment (Table S1). This is likely because the forest floor acts as a buffer for changes in temperature and precipitation (Sayer, 2006), and in addition to greater exposure of the soil surface in the L− plots, higher fine root biomass in the L− plots compared to the controls (Table S2) could also have contributed to altered sensitivity of soil respiration to soil temperature (Boone, Nadelhoffer, Canary, & Kaye, 1998). Similarly, soil respiration in the L− plots at Gigante was more strongly related to changes in soil water content than in CT and L+ plots with an intact litter layer, most likely because microbes and roots of bare soils are more sensitive to rapid changes in soil water conditions under constant soil temperature.

### 4.2 Litterfall seasonality, soil respiration, and priming effects

Although the seasonal patterns in total soil respiration and soil C release by priming effects at our study sites were largely explained by soil water content and soil temperature, litterfall also made an important contribution to temporal variation in soil respiration and priming effects in both forests. The vast majority (c. 75%) of litterfall at Wytham occurred within three to four months after the end of the growing season, whereas there was a smaller peak in litterfall during the dry season at Gigante and leaf abscission was otherwise evenly distributed throughout the year. Nonetheless, we observed positive feedbacks of litterfall on temporal variation in soil respiration and priming effects at both sites. In the control treatments, peak soil respiration occurred c. 8 months after peak litterfall at Wytham and c. 4 months later at Gigante. Accordingly, our models of soil respiration were improved by including monthly litterfall with corresponding time‐lags (Table 1). The distinct time‐lags between sites reflects the differences in the rate of litter decomposition and soil organic C turnover between wet tropical and temperate climates. Based on measured mean decay rates of 0.69 for Wytham (Medina‐Barcenas, unpublished data) and 1.74 for Gigante (Sayer et al., 2006), the time‐lags indicate that litter inputs have the greatest influence on belowground respiration at around 50% mass loss (c. 45% at Wytham and c. 55% at Gigante).

We propose that distinct litterfall patterns and C turnover rates also contributed to the differences in the magnitude of treatment responses between sites, and to soil C release by priming effects. Due to the seasonal nature of litterfall at Wytham, the litter treatments were only applied during the months of peak litterfall, but they captured 75%–80% of the total annual litterfall. Accordingly, soil respiration increased immediately after the first application of litter but, more surprisingly, the effect of litter addition and the soil C release by priming persisted throughout the year. By contrast, although the litter treatments in the tropical lowland forest at Gigante also started during the period of peak litterfall in the dry season, the treatments only captured 45% of the total annual litterfall over the same timeframe (4 months), and we saw no response of soil respiration to the litter treatments during the first year of the study (Figure 3d). As we measured respiration from the mineral soil, changes in soil respiration in response to litter treatments are only likely to be detected when the treatments start to influence processes in the mineral soil. A similar delay in the effects of litter manipulation treatments on soil respiration in a previous experiment in the same tropical forest was attributed to low soil water content during the dry season, which limits decomposition processes and heterotrophic soil respiration (Sayer et al., 2007). However, cold winter temperatures would also limit decomposition processes at Wytham, whereas the smaller proportion of total annual litterfall transferred during the first few months of the experiment, in combination with the faster C turnover, could explain why we saw little effect of litter addition treatment during the first year at Gigante. In the tropics, rapid turnover of C in the surface litter layer likely results in a smaller proportion of litter‐derived C being incorporated into the soil and thus a smaller and more gradual influence of litter addition treatments. It is important to note that the greater response of soil respiration to litter addition treatments at Wytham persisted after the first year. The more immediate effect of treatments at Wytham could therefore reflect the importance of high seasonal litter C inputs to soil heterotrophs, whereas the slower rate of turnover would explain the greater influence of litter addition treatments at Wytham, as well as, the persistence of both treatment and priming effects throughout the year. The links between litterfall seasonality, C turnover rates, and priming effects suggests that more frequent extreme events (i.e., tree damage, changes in litterfall) expected under climate change, have the potential to significantly alter soil C dynamics (Sayer et al., 2011).

## 5 LIMITATIONS TO THE STUDY

The aim of our study was to compare and contrast the response of soil respiration to altered litter inputs in two distinct wooded ecosystems. Our approach allowed us to identify common patterns and differences in potential soil C release by priming, but the high heterogeneity and low degree of control in such large‐scale field studies preclude identification of specific mechanisms. Our estimates of soil C release by priming are based on differences in soil respiration among treatments, because there were no significant changes in microbial biomass or fine root biomass that would account for changes in soil respiration (Sayer et al., 2007; Supporting Information S2). However, we do not consider potential differences in root turnover or exudation, which could mitigate or amplify the effects of our litter manipulation treatments Lopez‐Sangil et al., 2017, as well as the differences in treatment responses between sites. Importantly, we currently lack fundamental insights into the potential influence of biotic factors such as rhizosphere processes and resource‐competition among organisms on soil C release by priming effects under global change. Nonetheless, the general patterns we identify here represent an important first step to identifying the wider relevance of priming effects in forest ecosystems.

## 6 CONCLUSIONS

To our knowledge, this is the first large‐scale experiment comparing the effects of altered aboveground litter inputs on soil respiration across distinct climatic zones. Our study demonstrates that although the occurrence of priming effects at our study sites largely tracked the seasonal dynamics of litterfall and soil respiration, the timing, frequency and magnitude of soil C release by priming were harder to predict. In contrast to our original hypothesis, soil C release by priming was more consistent and occurred more frequently in the temperate woodland, which may be a result of slower C turnover. Our results contribute to understanding in situ priming effects in different forest ecosystems, but much further work is needed to identify the underlying mechanisms.

## CONFLICT OF INTEREST

None declared.

## AUTHORS CONTRIBUTION

L.M.B. and B.C.T. conducted measurements on soil respiration, litter, and soil properties in Panama and L.L‐S., C.G., A.J.B., and C.B. conducted measurements on soil respiration, litter, and soil properties in the UK. L.M.B. and E.J.S. led the data analysis and interpretation. All authors provided input on the analyzes and interpretation of the results and L.M.B., L.L‐S., C.G., A.J.B., C.B., and E.J.S. contributed to writing the manuscript.
