# Supplementary material for: Distinct responses of soil respiration to experimental litter manipulation in temperate woodland and tropical forest
Source: Ecol Evol. 2018 Mar 13;8(7):3787–96. doi: 10.1002/ece3.3945 (PMC5901162; doi:10.1002/ece3.3945)
Supplement: Supplementary file 1 [file ECE3-8-3787-s001.docx]

### Supplementary Table S1.

### Relationships between soil respiration and soil temperature and water content.

### Showing the results of models describing the relationships between soil respiration (SR) and soil temperature (T_Soil_) and water content (SWC) in different litter manipulation treatments at Wytham Woods and Gigante Forest from December 2013 to November 2016, where L- is litter removal; CT is control; and L+ is litter addition (*n* = 35). Since soil temperature at Gigante Forest only varied by *c*. 5°C throughout the year, there was no relationship between soil respiration and soil temperature and it was not possible to determine a Q_10_ value for the site.

| *Wytham Woods* | | | a | | b | c | F | *p* | Q_10_ | R^2^ |
| --- | --- | --- | --- | --- | --- | --- | --- | --- | --- | --- |
|  |  |  |  | |  |  |  |  |  |  |
|  |  |  | | SR *vs.* T_Soil_; $SR=ae^{(bT_{Soil})}$ | | | | | |  |
| L- | | | 0.6219 | | 0.1280 |  | 291.6 | < .0001 | 3.60 | 0.78 |
| CT | | | 0.6842 | | 0.1182 |  | 352.1 | < .0001 | 3.26 | 0.83 |
| L+ | | | 1.0726 | | 0.1034 |  | 544.2 | < .0001 | 2.81 | 0.82 |
|  |  |  | | SR *vs.* SWC; $SR=aSWC+b$ | | | | | |  |
| L- | | | -0.1077 | | 6.9996 |  | 50.4 | < .0001 |  | 0.60 |
| CT | | | -0.1061 | | 7.2162 |  | 50.2 | < .0001 |  | 0.60 |
| L+ | | | -0.0856 | | 6.9815 |  | 28.7 | < .0001 |  | 0.47 |
| *Gigante Forest* | | | | | | | | | |  |
|  |  |  | | SR *vs.* SWC; $SR=a+bSWC+c{SWC}^{2}$ | | | | | |  |
| L- | | | -3.0821 | | 0.5470 | -0.0086 | 39.3 | < .0001 |  | 0.74 |
| CT | | | -3.7097 | | 0.6190 | -0.0098 | 27.5 | < .0001 |  | 0.66 |
| L+ | | | -3.6783 | | 0.6006 | -0.0091 | 10.4 | 0.0004 |  | 0.43 |

**Supplementary Material S2.**

**Microbial and fine root biomass**

We collected soil samples to quantify microbial biomass during the growing season in 2015 and 2016 at both sites. We took nine soil cores from random locations within each plot and pooled them to make one composite sample per plot. All soil cores were collected at 0 - 10 cm depth using a 2-cm or 3-cm diameter punch-corer. We determined microbial biomass C and N by fumigation extraction (Vance *et al.*, 1987 with modifications by Jones & Willett, 2006) using paired subsamples of 8-g fresh weight. Briefly, one sample was exposed to ethanol-free chloroform for 24-h in the dark (fumigated samples) and both soil samples were shaken in 40-ml 0.5 M K_2_SO_4_, followed by centrifugation and filtration. Total organic C and N in the extracts were analysed on a TOC-L combustion analyser coupled with a TNM-L unit (Shimadzu Corp, Kyoto, Japan) and microbial biomass C and N were estimated from the difference between fumigated and unfumigated samples (expressed as oven-dry mass).

We determined fine root biomass (diameter ≤ 2 mm) in the mineral soil at both sites in August 2015 and August - September 2016. In 2015, five (Gigante) or six (Wytham) 4.8-cm diameter soil cores were taken at 0-10 cm depth at random locations within the inner 15-m × 15-m of each plot. In 2016, six 3.2-cm diameter soil cores per plot were collected at both sites. Fine roots were carefully removed from the cores by sieving and washing, dried at 40°C in Panama and 60°C in the UK and weighed.

We assessed the effects of litter manipulation on microbial biomass C and root biomass for each site using linear mixed effects models with treatment as fixed effects and block and time as a random effect. Except at Wytham for fine root biomass (AIC = 346.0; χ^2^ = 2.02; n = 30), none of the models were a significantly better fit compared to the corresponding null model, indicating no effect of treatment on microbial biomass C or fine root biomass (at Gigante Forest) among treatments at either site at any time.

**Supplementary Table S2** showing mean changes in microbial biomass carbon (µg g^-1^) and fine root biomass (< 2 mm; g m^-2^) in the mineral soil at 0 - 100 mm in response to litter manipulation treatments between 2015 and 2016 in temperate woodland (Wytham Woods) in the UK, and lowland tropical forest (Gigante Forest), in Panama; L- is litter removal; CT is control; and L+ is litter addition (*n* = 5).

|  | Wytham Woods | | | Gigante Forest | | |
| --- | --- | --- | --- | --- | --- | --- |
|  | L- | CT | L+ | L- | CT | L+ |
| *Microbial biomass C (µg g^-1^)* | *Mean ±STD* | | | *Mean ±STD* | | |
| 2015 | 293.5 *±*27.5 | 318.5 *±*27.3 | 318.6 *±*24.9 | 351.8 *±*36.6 | 355.4 *±*35.5 | 372.8 *±*36.1 |
| 2016 | 298.1 *±*60.8 | 315.3 *±*35.4 | 380.3 *±*39.6 | 192.2 *±*31.2 | 249.8 *±*41.3 | 260.6 *±*8.6 |
| *Fine root biomass*  *(g m^-2^)* |  |  |  |  |  |  |
| 2015 | 530.9 *±*35.5 | 384.7 *±*42.0 | 541.7 *±*60.1 | 246.6 *±*28.8 | 296.7 *±*36.8 | 329.9 *±*38.0 |
| 2016 | 470.4 *±*49.6 | 275.8 *±*16.9 | 340.5 *±*54.4 | 318.1 *±*29.9 | 283.6 *±*35.9 | 269.8 *±*43.3 |
